# Influence of secondhand smoke exposure on the retinal vasculature of children in Hong Kong

**DOI:** 10.1038/s43856-023-00389-4

**Published:** 2023-10-26

**Authors:** Carol Y. Cheung, Xiu Juan Zhang, Hei-Nga Chan, Yuzhou Zhang, Vincent L. Yuen, Wynne Hsu, Mong Li Lee, Dejiang Xu, Jason Wong, Fang Yao Tang, Kai Wai Kam, Alvin Young, Mandy P. Ng, Patrick Ip, Li Jia Chen, Tien Y. Wong, Chi Pui Pang, Clement C. Tham, Jason C. Yam

**Affiliations:** 1 Department of Ophthalmology and Visual Sciences, The Chinese University of Hong Kong, Hong Kong SAR, China; 2 School of Computing, National University of, Singapore, Singapore; 3 Department of Ophthalmology and Visual Sciences, Prince of Wales Hospital, Hong Kong SAR, China; 4 Department of Paediatrics and Adolescent Medicine, University of Hong Kong, Hong Kong SAR, China; 5 Hong Kong Hub of Paediatric Excellence, The Chinese University of Hong Kong, Hong Kong SAR, China; 6 Singapore Eye Research Institute, Singapore National Eye Center, Singapore, Singapore; 7 Tsinghua Medicine, Beijing Tsinghua Changgung Hospital, Tsinghua University, Beijing, China; 8 Hong Kong Eye Hospital, Kowloon, Hong Kong SAR, China; 9 Department of Ophthalmology, Hong Kong Children’s Hospital, Hong Kong SAR, China

**Keywords:** Population screening, Paediatric research

## Abstract

**Background:**

A recent prospective demonstrated that cardiovascular risk factors in early childhood were associated with later cardiovascular events. However, the impact of secondhand smoke (SHS) on children is unclear. The aims of this study is to determine the effects of SHS exposure on the retinal vasculature of children.

**Methods:**

This is a population-based cross-sectional study of children aged 6 to 8 years. All participants received comprehensive ophthalmic examinations and retinal photography. Data on SHS exposure was derived from a validated questionnaire. A validated deep-learning system was used to automatically estimate retinal arteriolar and venular calibers from retinal photographs. Associations of quantitative retinal vessel caliber values with SHS exposure, number of smokers in the household, and total number of cigarettes smoked were determined by analyses of covariance (ANCOVA) after adjusting for potential confounders. Test of trend was determined by treating categorical risk factors as continuous ordinal variables.

**Results:**

Here we show children exposed to SHS have wider retinal arteriolar (CRAE 152.1 µm vs. 151.3 µm, *p* < 0.001) and venular (CRVE 216.7 µm vs. 215.5 µm, *p* < 0.001) calibers compared to those in smoke-free homes, after adjustment for different factors. Wider arteriolar and venular calibers are also associated with increasing number of smokers in the family (*p* trend < 0.001) and more cigarettes smoked among family smokers (*p* trend<0.001).

**Conclusions:**

Exposure to SHS at home is associated with changes in retinal vasculature among children. This reinforces the adverse effect of secondhand smoking around children though further research incorporating comprehensive assessment of potential confounders is necessary.

## Plain Language Summary

Exposure to secondhand smoke can be harmful, particularly for our heart and lung health as adults. However, the impact of secondhand smoke on children is less clear. Here, we looked at the effects of secondhand smoke exposure on vessels within children’s eyes. The health of these vessels is a potential indicator of overall eye health and is also associated with cardiovascular disease. Pictures were taken of children’s eyes and analyzed using a computer program. We looked at the association between vessel measurements in the eye and how much secondhand smoke the children are exposed to. We observed differences in the vessels in children exposed to secondhand smoke, compared to those from smoke-free homes. These findings indicate that secondhand smoke may affect the health of children’s eyes and highlight the need to promote smoke-free home environments.

## Abstract (web summary)

Cheung, Zhang et al. examine the association between secondhand smoke exposure and changes in the retinal vasculature among children aged 6 to 8, in a population-based cohort study. Children exposed to secondhand smoke have wider retinal arteriolar and venular calibers compared to those in smoke-free homes.

## Introduction

Secondhand smoke (SHS) is a major public health problem, accounting for ~880,000 deaths globally each year^1–3^. SHS contains the same chemical substances that exert pervasive and detrimental effects^4–6^, and in adults is associated with risks of a range of cardiovascular and respiratory diseases^7–9^. However, the impact of SHS on children is less well known. In particular, the longer-term effect of SHS during childhood on cardiovascular and metabolic health in adulthood is unclear. Furthermore, exposure to SHS is highly prevalent, affecting possibly 40% of children worldwide^2^. COVID-19 lockdowns have increased the problem as children stay home longer and may have increased SHS exposure^10^.

The retinal vasculature, which is accessible to direct non-invasive imaging, shares similar anatomical and physiological characteristics with the vasculatures of many other organs in the body, and changes in the retinal vasculature reflect pathological processes in the systemic circulation^11–16^. Large population studies have demonstrated that variation in caliber of retinal vessels, usually measured from retinal photographs, is associated with a range of cardiovascular disease (CVD) risk factors including hypertension, diabetes and smoking^17–23^. In particular, smoking is associated with retinal arteriolar and venular caliber dilation^17–24^, and these retinal vessel caliber changes are predictive of cardiovascular and cerebrovascular diseases and mortality^25–34^. There are fewer studies of retinal vascular changes in children, but retinal vessel dilation has also been associated with higher blood pressure, lower birthweight and higher body mass index, suggesting that the impacts of cardiovascular risk factors on the retinal vasculature occur in early life and track towards adulthood even before the onset of overt CVDs^35–41^.

Previous studies have predominantly focused on the effects of firsthand tobacco smoking on adult retinas;^17–24^ to the best of our knowledge, the effects of SHS exposure on the retinal vasculatures of children have not yet been investigated. Furthermore, we have developed and validated a fully automated artificial intelligence (AI) deep-learning system to estimate retinal vessel calibers which provides unique information on vascular health^42^. In this current study, we evaluated the relationship of SHS on the retinal vasculature in children. We hypothesize that if SHS exposure is toxic^4,9,43^, SHS exposure will affect children’s retinal vascular health reflected by retinal arteriolar and venular dilation, similar to retinal vessel changes associated with smoking in adults^17–24^. We test this hypothesis among schoolchildren aged 6 to 8 years from a population-based study and use the validated deep-learning system for measuring retinal vessel calibers from retinal photographs^42^. In this study, children exposed to SHS has wider retinal arteriolar (CRAE 152.1 µm vs. 151.3 µm, *p* < 0.001) and venular (CRVE 216.7 µm vs. 215.5 µm, *p* < 0.001) calibers compared to those in smoke-free homes, after adjustment with different factors. Wider arteriolar and venular calibers are also associated with increasing number of smokers in the family (*p* trend<0.001) and more cigarettes smoked among family smokers (*p* trend<0.001).

## Methods

### Study participants

We conducted a population-based cross-sectional study. All participants were recruited from the Hong Kong Children Eye Study (HKCES), a population-based study of eye conditions among schoolchildren aged 6 to 8 years in Hong Kong, China^26,41^. They all took part in comprehensive ophthalmic examinations, physical examinations, and standardized interviews. This study excluded children who had congenital malformations, prior ocular trauma, history of ocular surgery, and ocular disorders except mild refractive errors. Children incapable of cooperating were also excluded.

### Questionnaires

The parents or legal guardians of participating children were asked to complete questionnaires in person or over the phone regarding the smoking habits of themselves and other family members in the same household^44–47^. Such information was obtained through the following questions, (1) has the mother smoked at home after the child was born? How long has she been smoking, and how many cigarettes a day; (2) Has the father smoked at home after the child was born? How long has he been smoking, and how many cigarettes a day; and (3) Have other family members smoked at home after the child was born? How long have they each been smoking, and how many cigarettes a day?

Parents or legal guardians also provided information on smoking habits for all family members living together with the participating children, including grandparents, siblings, and other relatives, after verifying with particular family members. Smoking outside of home was not counted toward SHS exposure. Ex-smokers who had quit before the child was born were considered equivalent to non-smokers in the analysis.

Children were allocated into the SHS group if one or more family members had smoked at home after they were born. The questionnaires documented the number of cigarettes smoked per day for individual family members, with the total number of cigarettes smoked at home per day by all smokers living with the children to determine their quantity of SHS exposure. The children’s lifestyle was also recorded, including their daily routines and living environments, plus information of family income, parental education levels, parental myopia, medical conditions, children’s birth history, their past and current medical history, and thorough family history of eye disorders. All questionnaires were completed with assistance from trained staff, who confirmed missing or uncertain information through additional phone calls after on-site interviews.

### Retinal photography

Retinal photographs were taken using a digital fundus camera (TRC-50DX; Topcon, Tokyo, Japan, with a color sensor resolution of 12.3MP and image sensor of 1.1 inch) after pupil dilation using 1% cyclopentolate and 1% tropicamide under a standardized setting. Two retinal photographs, centered at the optic disc and fovea, respectively, were obtained for each eye.

### Measurement of retinal vessel calibers

The Singapore I Vessel Analyzer-Deep-Learning System (SIVA-DLS), is a fully automated AI-deep learning based system to measure retinal vessel caliber^42^. Photographs centered at the right eye optic disc was assessed; if that photograph was ungradable, measurements would be performed on the corresponding photograph for the left eye.

The SIVA-DLS^42^ estimated the values for retinal arteriolar caliber, referred to as central retinal artery equivalent (CRAE) and retinal venular caliber, referred to as the central retinal vein equivalent (CRVE) from the retinal photographs. It uses a convoluted neural network to estimate the values in the region within 0.5 to 2.0 disc diameters away from the optic disc margin. Heatmaps were generated to highlight the regions it focused on to calibrate its CRAE and CRVE predictions. After training and validation, SIVA-DLS was externally tested using a large, multi-ethnic, multi-country dataset of >70,000 retinal photographs from 15 datasets, including one from the HKCES^42^.

Prior to caliber estimation, initial gradability of the photograph was assessed by SIVA-DLS. Those with poor image quality or unreliable caliber prediction by SIVA-DLS were excluded from analysis (see examples in Supplementary Figure 1), such as images with high reflection (e.g., due to thicker retinal nerve in children that affected the visibility of retinal vessels), blurry images (e.g., due to axial movement), artifacts or images with poor centration (i.e., the optic nerve head not being centered).

### Other examinations

Trained ophthalmologists conducted complete ocular examinations for each participant, including examinations of the anterior segment, posterior segment, and ocular motility. Refraction was measured before and after cycloplegia using an autorefractor (Nidek ARK-510A, Gamagori, Japan); spherical equivalent refraction was calculated as the algebraic sum of the sphere value and half of the cylinder value. Ocular axial length was evaluated using an interferometric device (IOL Master; Carl Zeiss Meditec AG, Jena, Germany).

Blood pressure (BP) was measured in the seated position after a 5-minute rest using a digital autonomic BP monitor (Vital Signs Monitor; Heal Force Bio-Meditech, Shanghai, China), with an appropriate cuff size for accurate measurements. Three measurements were taken, with the average result taken for subsequent analysis. Both systolic (SBP) and diastolic blood pressure values were classified into height-specific BP cutoff values according to the American Academy of Pediatrics guidelines^48^. Children with BP within the 90^th^ percentile were classified as normotensive, between the 90th and 95th percentiles as elevated BP, and above the 95th percentile as hypertensive. The term “hypertensive” was used instead of “hypertension” because BP measurement in a single visit cannot diagnose hypertension. Body height and weight were measured using a professional integrated set (seca; Hamburg, Germany). Children were categorized as normal, overweight, and obese using the age- and sex-specific BMI cutoff values from the International Obesity Task Force guidelines^49^.

### Statistical analysis

Demographic results are reported as means or percentages, with differences tested by independent *t*-tests, analysis of variance tests, and chi-square tests. Analysis of covariance (ANCOVA) tests were used to estimate mean CRAE and CRVE values at presence or absence of smokers at home. The ANCOVAs also examined cross-sectional relationships between retinal vessel calibers and SHS exposure at home. Model 2 adjusted for age, sex, BMI and AL, Model 3 added mean arterial pressure, and Model 4 added family income, parental education level and parental myopia as factors. Model 5 adjusted for all the factors considered in Model 4 as well as a fellow vessel caliber to avoid biased results and minimize potential confounding from the fellow caliber^50^.For example, CRVE would be added to calculations of the effect of SHS on CRAE, and vice versa. Significant values for trends were analyzed by treating (0, 1, 2 or more) and the number of cigarettes smoked per day (none, ten or less, over 10) as continuous ordinal variables. Subgroup analyses stratified by BP, sex, and BMI were tested for potential effect modifications from these factors on associations between SHS and retinal vessel calibers by including cross-product interaction terms as independent variables in the ANCOVA. All statistical analyses were performed using SPSS (version 24; IBM, Armonk, NY). Significance levels of *p* < 0.05 were used.

### Ethical considerations

The study adhered to the Declaration of Helsinki, with the study protocol being approved by the Ethics Committee Board of the Chinese University of Hong Kong (CREC 2015.033). All children and their parents or legal guardians signed written informed consent forms upon their participation in the study.

### Reporting summary

Further information on research design is available in the Nature Portfolio Reporting Summary linked to this article.

## Results

A total of 11,141 children underwent ophthalmic investigations in this study, among them 779 were excluded for further investigations due to poor image quality or unreliable caliber prediction by SIVA-DLS, leaving 10,362 (93.0%) subjects for final analysis. The mean age ± standard deviation was 7.59 ± 1.08 years, 48.1% were girls, and 51.9% were boys. Among them, 34.6% were exposed to SHS, and 65.4% were not. There were no significant differences in age, sex, BP, ocular axial length, spherical equivalent, outdoor activity time, or diopter hours between the two groups. However, children exposed to SHS were more likely to have higher BMI, lower family income, lower parental education level, and lower parental myopia rates (*p* < 0.001) **(**Table 1). The demographics of the current study was similar to our first phase population-based study. (Supplementary Table 1)

**Table 1:** Baseline demographics and characteristics of participants.

	No Smoking Exposure (*n* = 6688)	Smoking Exposure (*n* = 3674)	*P* values
Age, years	7.59 (1.09)	7.56 (1.08)	0.27
BMI, kg/m^2^	16.07 (2.77)	16.40 (2.95)	**<0.001**
Axial length, mm	23.15 (0.94)	23.12 (0.95)	0.12
Spherical equivalent, D	0.15 (1.50)	0.17 (1.54)	0.67
Outdoor activity time, hours per day	1.40 (0.53)	1.41 (0.55)	0.70
^b^Diopter-hours	11.11 (2.96)	11.15 (3.11)	0.52
Systolic blood pressure, mmHg	101.94 (11.50)	101.71 (11.37)	0.36
Diastolic blood pressure, mmHg	64.98 (9.12)	64.68 (8.95)	0.12
Mean arterial pressure, mmHg	77.30 (8.92)	77.02 (8.85)	0.15
Sex, No. (%)			
Boys	3463 (51.8)	1915 (52.1)	0.77
Girls	3225 (48.2)	1759 (47.9)	
Family income per month, No. (%)			
≤ ^a^HK$20,000	1679 (25.1)	1576 (42.9)	**<0.001**
> ^a^HK$20,000	5009 (74.9)	2098 (57.1)	
Parents’ education level, No. (%)			
Father			
High school or lower	3805 (56.9)	3152 (85.8)	**<0.001**
Bachelor degree or higher	2883 (43.1)	522 (14.2)	
Mother			
High school or lower	4267 (63.8)	3185 (86.7)	**<0.001**
Bachelor degree or higher	2421 (36.2)	489 (13.3)	
Parents’ myopia, No. (%)			
Father			
No	3217 (48.1)	2355 (64.1)	**<0.001**
Yes	3471 (51.9)	1319 (35.9)	
Mother			
No	2963 (44.3)	1977 (53.8%)	**<0.001**
Yes	3725 (55.7)	1697 (46.2%)	

Bold values indicate statistical significance *p* < 0.05.

Statistical significance was tested using independent *t* test; χ2 test and Fisher exact test were used to test the group difference for categorical data.

BMIs were calculated as weight in kilograms divided by height in meters squared.

^a^US $2551.10.

^b^“Diopter-hours” weighs various near activities and viewing distances to determine a comprehensive value of near work exposure. A formula for calculating diopter-hours was defined as: (hours spent studying + hours spent reading for pleasure)*3 + (hours spent playing video games or working on the computer at home)*2 + (hours spent watching television) *1

**Fig. 1:**
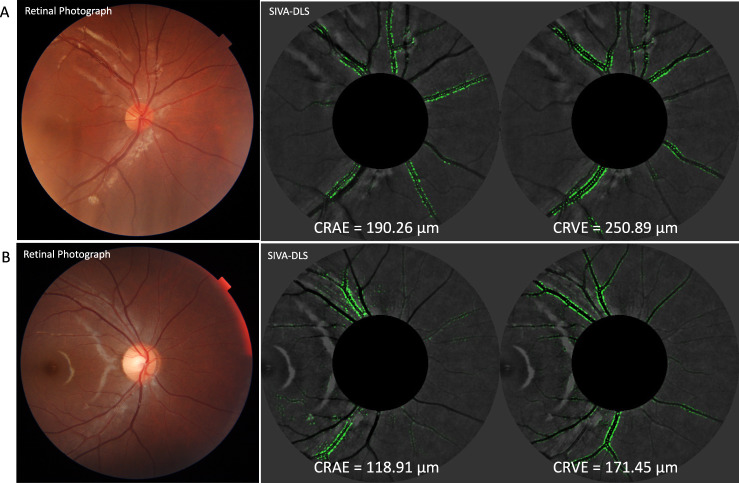
Comparison of retinal photographs and SIVA-DLS heatmaps in children with varied SHS Exposure. The figure displays retinal photographs of children with (**A**) SHS exposure and (**B**) without SHS exposure on the left. On the right, SIVA-DLS heatmaps highlight arteriolar and venular boundaries, used to predict CRAE and CRVE, respectively. SIVA-DLS Singapore I Vessel Analyzer-Deep-Learning System, CRAE central retinal artery equivalent, CRVE central retinal vein equivalent.

Children with SHS exposure had larger CRAE (152.9 µm vs. 151.0 µm, *p* < 0.001) and CRVE (217.9 µm vs. 215.0 µm, *p* < 0.001) compared to children without SHS exposure after adjusting for age, sex, parental education level, family income, mean arteriolar BP, BMI, and axial length (Table 2, Model 4). The associations remained (CRAE 152.1 µm vs. 151.3 µm, *p* < 0.001; CRVE 216.7 µm vs. 215.5 µm, *p* < 0.001) after fellow vessel caliber was adjusted. (Table 2, Model 5) Fig. 1 shows the estimations of CRAE and CRVE computed by SIVA-DLS on retinal photographs of two children, one SHS exposure and one without. The heatmaps generated by SIVA-DLS highlighted the boundaries for arterioles and venules used to predict CRAE and CRVE, respectively.

**Table 2:** Associations between exposure to smoking and retinal vessel calibers.

	No smoking exposure (*n* = 6688)	Smoking exposure (*n* = 3674)	Mean difference (95%CI)	*P* values
CRAE, µm		Mean (95% CI)		
Model 1	151.0 (150.7, 151.3)	152.9 (152.4, 153.3)	1.9 (1.3, 2.4)	**<0.001**
Model 2	151.0 (150.7, 151.4)	152.9 (152.4, 153.3)	1.8 (1.3, 2.4)	**<0.001**
Model 3	151.0 (150.7, 151.4)	152.9 (152.4, 153.3)	1.8 (1.3, 2.4)	**<0.001**
Model 4	150.9 (150.5, 151.3)	152.9 (152.3, 153.4)	2.0 (1.3, 2.6)	**<0.001**
Model 5	151.3 (151.0, 151.6)	152.1 (151.8, 152.5)	0.8 (0.4, 1.2)	**<0.001**
CRVE, µm				
Model 1	215.1 (214.6, 215.6)	217.8 (217.1, 218.4)	2.7 (1.9, 3.5)	**<0.001**
Model 2	215.1 (214.6, 215.6)	217.8 (217.1, 218.4)	2.7 (1.9, 3.5)	**<0.001**
Model 3	215.1 (214.6, 215.6)	217.8 (217.1, 218.4)	2.7 (1.9, 3.5)	**<0.001**
Model 4	215.0 (214.4, 215.5)	217.8 (217.0, 218.6)	2.9 (2.0, 3.8)	**<0.001**
Model 5	215.5 (215.1, 215.9)	216.7 (216.1, 217.2)	1.2 (0.6, 1.8)	**<0.001**

Bold values indicate statistical significance *p* < 0.05.

Model 1: not adjusted; Model 2: adjusted for age, sex, body mass index, axial length; Model 3: adjusted for age, sex, body mass index, axial length, and mean arterial pressure; Model 4: adjusted for age, sex, body mass index, axial length, global RNFL, mean arterial pressure, family income, parental education level, and parental myopia; Model 5: adjusted for age, sex, body mass index, axial length, mean arterial pressure, family income, parental education level, parental myopia, and fellow vessel caliber.

*CRAE* central retinal artery equivalent, *CRVE* central retinal vein equivalent, *CI* confidence interval.

Study participants were further classified into groups according to the number of smokers in their families: none (*n* = 6688), 1 (*n* = 3341), and two or more smokers (*n* = 333). The number of smokers in the family was associated with retinal vessel caliber among children: with more smokers in their families the children had larger CRAE and CRVE (all *p*_trend_ < 0.001) (Table 3). Total cigarettes smoked by family smokers were associated with retinal vessel caliber (Table 4). When categorized into groups of 0 (*n* = 6688), 10 or fewer (*n* = 1520), and over ten cigarettes per day (*n* = 2154), wider CRAE and CRVE were both associated with an increased number of cigarettes smoked by the family (all *p*_trend_ < 0.001).

**Table 3:** Associations between number of smokers and retinal vessel calibers.

	No smoker (*n* = 6688)	1 smoker (*n* = 3341)	≥2 smokers (*n* = 333)	*P* values for trend
CRAE, µm		Mean (95%CI)		
Model 1	151.0 (150.7, 151.3)	152.6 (152.1, 153.1)	155.8 (154.3, 157.3)	**<0.001**
Model 2	151.0 (150.7, 151.4)	152.6 (152.1, 153.1)	155.9 (154.4, 157.4)	**<0.001**
Model 3	151.0 (150.7, 151.4)	152.5 (152.1, 153.0)	156.1 (154.6, 157.6)	**<0.001**
Model 4	150.9 (150.5, 151.3)	152.6 (152.1, 153.2)	155.6 (154.0, 157.2)	**<0.001**
Model 5	151.3 (151.1, 151.6)	152.0 (151.6, 152.4)	153.7 (152.6, 154.8)	**<0.001**
CRVE, µm				
Model 1	215.1 (214.6, 215.6)	217.4 (216.7, 218.0)	221.7 (219.5, 223.8)	**<0.001**
Model 2	215.1 (214.6, 215.6)	217.4 (216.7, 218.1)	221.9 (219.7, 224.0)	**<0.001**
Model 3	215.1 (214.6, 215.6)	217.4 (216.7, 218.0)	222.1 (219.9, 224.2)	**<0.001**
Model 4	215.0 (214.4, 215.5)	217.5 (216.7, 218.3)	221.6 (219.3, 223.9)	**<0.001**
Model 5	215.5 (215.1, 215.9)	216.5 (216.0, 217.1)	218.4 (216.8, 219.9)	**<0.001**

Bold values indicate statistical significance *p* < 0.05.

Model 1: not adjusted; Model 2: adjusted for age, sex, body mass index, axial length; Model 3: adjusted for age, sex, body mass index, axial length, and mean arterial pressure; Model 4: adjusted for age, sex, body mass index, axial length, global RNFL, mean arterial pressure, family income, parental education level, and parental myopia; Model 5: adjusted for age, sex, body mass index, axial length, mean arterial pressure, family income, parental education level, parental myopia, and fellow vessel caliber.

*CRAE* central retinal artery equivalent, *CRVE* central retinal vein equivalent, *CI* confidence interval.

**Table 4:** Associations between quantity of smoking and retinal vessel calibers.

	0 cigarette (*n* = 6688)	<= 10 cigarettes (*n* = 1520)	>10 cigarettes (*n* = 2154)	*P* values for trend
CRAE, µm		Mean (95%CI)		
Model 1	151.0 (150.66, 151.34)	152.22 (151.51, 152.92)	153.32 (152.73, 153.91)	**<0.001**
Model 2	151.0 (150.68, 151.35)	152.23 (151.53, 152.94)	153.37 (152.78, 153.96)	**<0.001**
Model 3	151.0 (150.70, 151.37)	152.17 (151.47, 152.87)	153.33 (152.74, 153.92)	**<0.001**
Model 4	150.9 (150.52, 151.32)	152.17 (151.39, 152.95)	153.39 (152.72, 154.07)	**<0.001**
Model 5	151.3 (151.05, 151.59)	151.79 (151.27, 152.32)	152.37 (151.92, 152.83)	**<0.001**
CRVE, µm				
Model 1	215.1 (214.6, 215.6)	216.8 (215.8, 217.8)	218.4 (217.6, 219.3)	**<0.001**
Model 2	215.1 (214.6, 215.6)	216.8 (215.8, 217.8)	218.5 (217.7, 219.4)	**<0.001**
Model 3	215.1 (214.6, 215.6)	216.7 (215.7, 217.7)	218.5 (217.7, 219.4)	**<0.001**
Model 4	215.0 (214.4, 215.5)	216.8 (215.7, 217.9)	218.6 (217.6, 219.5)	**<0.001**
Model 5	215.5 (215.1, 215.9)	216.2 (215.5, 217.0)	217.0 (216.3, 217.6)	**<0.001**

Bold values indicate statistical significance *p* < 0.05.

Model 1: not adjusted; Model 2: adjusted for age, sex, body mass index, axial length; Model 3: adjusted for age, sex, body mass index, axial length, and mean arterial pressure; Model 4: adjusted for age, sex, body mass index, axial length, global RNFL, mean arterial pressure, family income, parental education level, and parental myopia; Model 5: adjusted for age, sex, body mass index, axial length, mean arterial pressure, family income, parental education level, parental myopia, and fellow vessel caliber.

*CRAE* central retinal artery equivalent, *CRVE* central retinal vein equivalent, *CI* confidence interval.

Subgroup analysis was conducted for the effects of SHS exposure at home stratified by sex, BP, and BMI. The effect of SHS exposure on CRAE and CRVE values was consistent across categories for sex, BP, and BMI (Supplementary Figs. 2, 3).

## Discussion

Exposure to SHS is highly prevalent worldwide and may have increased during the COVID-19 lockdown as children stay home longer. The longer-term impact of SHS during childhood on future cardiovascular and metabolic health in adulthood is unclear. Results of our study showed retinal vessel dilation in children with SHS exposure. A greater number of smokers and a greater quantity of SHS exposure in the household were associated with wider retinal vessel calibers in children in a dose-dependent manner. We found this association in all subgroups of children, regardless of BP or body weight.

The retinal blood vessels provide unique information on vascular health. In the current study, we showed that children with SHS exposure were likely to have retinal vessel dilation. Our results in children were compared to what is known about retinal vessel changes in adults. Retinal vessel dilation is also seen in reported population-based and clinical studies in older adults associated with firsthand smoking^17–24^. For example, when comparing current smokers with non-smokers, the Blue Mountain Eye Study found that mean retinal vessel caliber were higher among smokers by (CRAE 10.8 µm and CRVE 8.1 µm, respectively)^24^. The Multi-Ethnic Study of Atherosclerosis found higher mean values of 9.8 µm and 3.6 µm, respectively^23^. In addition, we found SHS exposure in our study was associated with a “dose-dependent pattern”. This is also in alignment with other population-based studies of firsthand smoking in adults^17–24^. Dilation of retinal arterioles^23,51^, and venules^23,51–53^, may reflect oxidative stress, endothelial dysfunction, and inflammation. Wider retinal arterioles may be associated with diabetes and higher level of plasma fibrinogen^23^, while wider retinal venules may also be associated with coronary heart disease^31,54,55^, stroke^27,28,54^, atherosclerosis^54^, diabetes^23^, obesity^23^, and dyslipidemia^23^. Our results, therefore, provide additional evidence to support the World Health Organization’s recommendation that SHS exposure in children is detrimental to metabolic health in longer term^56^.

While our study was not designed to determine the underlying mechanisms, we speculate that retinal vessel dilation with SHS may be related to inflammation^24,57–60^, since tobacco smoking stimulates chronic low-level inflammation and activates leukocytes to disrupt the vascular endothelial surface that leads to venular dilation^24,60^. Another potential mechanism may be beta-adrenergic receptor activation;^19,61,62^ since nicotine causes beta-adrenoceptor-mediated vasodilation^62^. Other possible mechanisms include nitric oxide production^19,61,62^, ATP-sensitive potassium channel activation^62^, tissue oxygenation^63^, and endothelial dysfunction^9,24,64,65^.

Our findings highlight the importance of considering retinal vessel changes in children as potential markers of systemic vascular health^66–68^. A recent prospective cohort study also showed that cardiovascular risk factors in early childhood were associated with adult cardiovascular events and death from cardiovascular causes^69^. In addition, children who grow up in smoking families are more likely to become smokers themselves^70,71^. Reducing SHS in family exerts an indirect effect on the primary objectives of preventing smoking and enhancing protection of public health. Overall, to prevent such hazards to children, there should be stringent tobacco control policies and specific intervention programs targeted at parents and people living with young children.

Strengths of the present study is the inclusion of a large, unselected, and population-based sample of over 10,000 children, largely free of retinal and systemic vascular diseases. In addition, we used a deep-learning-based algorithm (SIVA-DLS) that automatically estimated the values of retinal vessel calibers from retinal photographs^42^. There are limitations in this study. Firstly, the cross-sectional study design limited the exploration of the causal and temporal relationships that retinal vascular parameters may have with cardiovascular and ocular factors. For example, the association between smoking and wider retinal vessels could be a transient one, mediated by carbon monoxide binding to hemoglobin. We are currently conducting follow-up studies on longitudinal changes in retinal vasculature resulting from SHS. Another limitation is the questionnaire, which only offered a snapshot of children’s exposure to SHS through their daily interactions with family members, which may not represent the typical behaviors of smokers in the household environment where children spend a lot of their time^72^. Of note, the reliance on self-reported smoking habits may be subject to not only the recall bias but also the social desirability bias, potentially leading to underreporting of the actual extent of smoking behavior among participants. We recommend conducting urinary cotinine measurements to provide objective and robust evidence of SHS exposure in future studies^73–76^. Thirdly, we did not adjust for some other potential cofounders, such as active and passive maternal smoking during pregnancy. Nevertheless, after excluding those with in utero exposure to maternal smoking, our sensitivity analysis showed the results remained largely similar (supplementary Table 2). The unmeasured confounding factors related to socioeconomic status and other environmental influences, such as nutritional status, physical activities, sleep quality, stress, medications, allergies, and air pollution were not included in this study. These factors have the potential to impact the observed associations. Further studies and mathematical modeling are warranted to clarify the relationships. In addition, we only included axial length in the statistical models which was indirectly adjusted for fundus magnification.

## Conclusions

In conclusion, our study found an association between SHS exposure at home and retinal vessel dilation in children. While caution is needed in interpreting these findings as causal due to potential unmeasured confounding factors, the results reinforce the importance of reducing children’s exposure to SHS and promoting smoke-free environments. Further research considering socioeconomic and environmental factors is necessary to enhance our understanding of the health risks associated with SHS exposure in children.

### Supplementary information

Supplementary Material

Reporting Summary

## Data Availability

The main data supporting the results in this study are available within the paper and its Supplementary Information. The deidentified individual-participant data and data on the evaluation of retinal photographs used in the SIVA-DLS are available on request from the corresponding author due to consent. (email address: yamcheuksing@cuhk.edu.hk)

## Code availability

The custom code is currently available only on request because it is under a patent examination process.
