# Supplementary material for: Influence of secondhand smoke exposure on the retinal vasculature of children in Hong Kong
Source: Commun Med (Lond). 2023 Oct 26;3:155. doi: 10.1038/s43856-023-00389-4 (PMC10603154; doi:10.1038/s43856-023-00389-4)
Supplement: Supplementary file 1 — Supplementary Material [file 43856_2023_389_MOESM1_ESM.pdf]

Supplementary Table 1. Demographics of Study Individuals

Characteristics	Current Study (N=10,362)	The HKCES – Phase 1 (N=4,257)	P value
Male-female ratio	1.08	1.10	0.58
Children's age (years)	7.59 (1.08)	7.62 (0.96)	0.12
Spherical Equivalent (D)	0.16 (1.52)	0.14 (1.59)	0.48
Axial Length (mm)	23.14 (0.94)	23.15 (0.95)	0.56
Children myopia rate (%)	26.4%	25.0%	0.09
Low Family Income Rate (%)	31.4%	38.0%	<0.001*
Smoking Exposure Rate (%)	34.6%	33.5%	0.20

HKCES: Hong Kong Children Eye Study; D = diopter.
Unless otherwise noted, values are mean (SD).
Low family income was defined as household income lower than 20,000 HKD.

Supplementary Table 2. Associations between Exposure to Smoking and Retinal Vessel Calibers (exclude those subjects with maternal smoking during pregnancy N=98)

	**No smoking exposure (n=6,688)**	**Smoking exposure (n=3,576)**	**Mean difference (95%CI)**	**P-values**
**CRAE, μm**	Mean (95% CI)			
Model 1	151.0 (150.7, 151.3)	152.8 (152.3, 153.2)	1.8 (1.2, 2.4)	**<0.001**
Model 2	151.0 (150.7, 151.4)	152.8 (152.4, 153.3)	1.8 (1.2, 2.4)	**<0.001**
Model 3	151.0 (150.7, 151.4)	152.8 (152.3, 153.2)	1.7 (1.2, 2.3)	**<0.001**
Model 4	150.9 (150.5, 151.3)	152.8 (152.3, 153.3)	1.9 (1.3, 2.5)	**<0.001**
Model 5	151.3 (151.1, 151.6)	152.1 (151.7, 152.4)	0.7 (0.3, 1.1)	**<0.001**
**CRVE, μm**				
Model 1	215.1 (214.6, 215.6)	217.7 (217.0, 218.3)	2.6 (1.8, 3.4)	**<0.001**
Model 2	215.1 (214.6, 215.6)	217.7 (217.1, 218.4)	2.6 (1.8, 3.4)	**<0.001**
Model 3	215.1 (214.6, 215.6)	217.7 (217.0, 218.4)	2.6 (1.8, 3.4)	**<0.001**
Model 4	215.0 (214.4, 215.5)	217.8 (217.0, 218.5)	2.8 (1.9, 3.7)	**<0.001**
Model 5	215.5 (215.1, 215.9)	216.6 (216.1, 217.2)	1.1 (0.5, 1.7)	**<0.001**

CRAE: central retinal artery equivalent; CRVE: central retinal vein equivalent; CI: confidence interval.

Model 1: not adjusted; Model 2: adjusted for age, sex, body mass index, axial length; Model 3: adjusted for age, sex, body mass index, axial length and mean arterial pressure; Model 4: adjusted for age, sex, body mass index, axial length, global RNFL, mean arterial pressure, family income, parental education level and parental myopia; Model 5: adjusted for age, sex, body mass index, axial length, mean arterial pressure, family income, parental education level, parental myopia and fellow vessel caliber.

Supplementary Figure 1. (A)-(D) Examples of excluded retinal photographs due to poor image quality or unreliable caliber prediction by SIVA-DLS

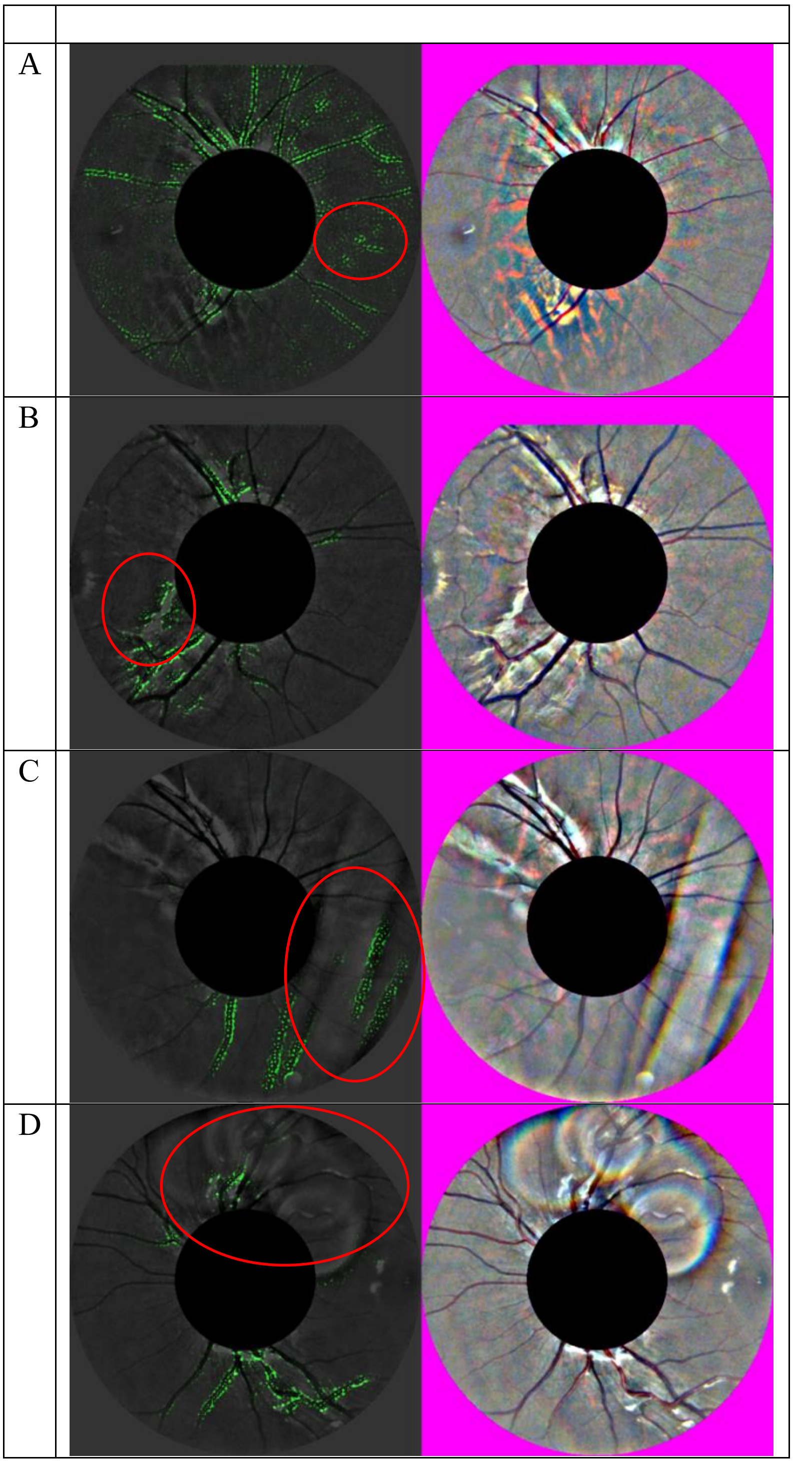

Supplementary Figure 2. Data Distribution of Retinal Vascular Caliber Stratified by Smoking Exposure

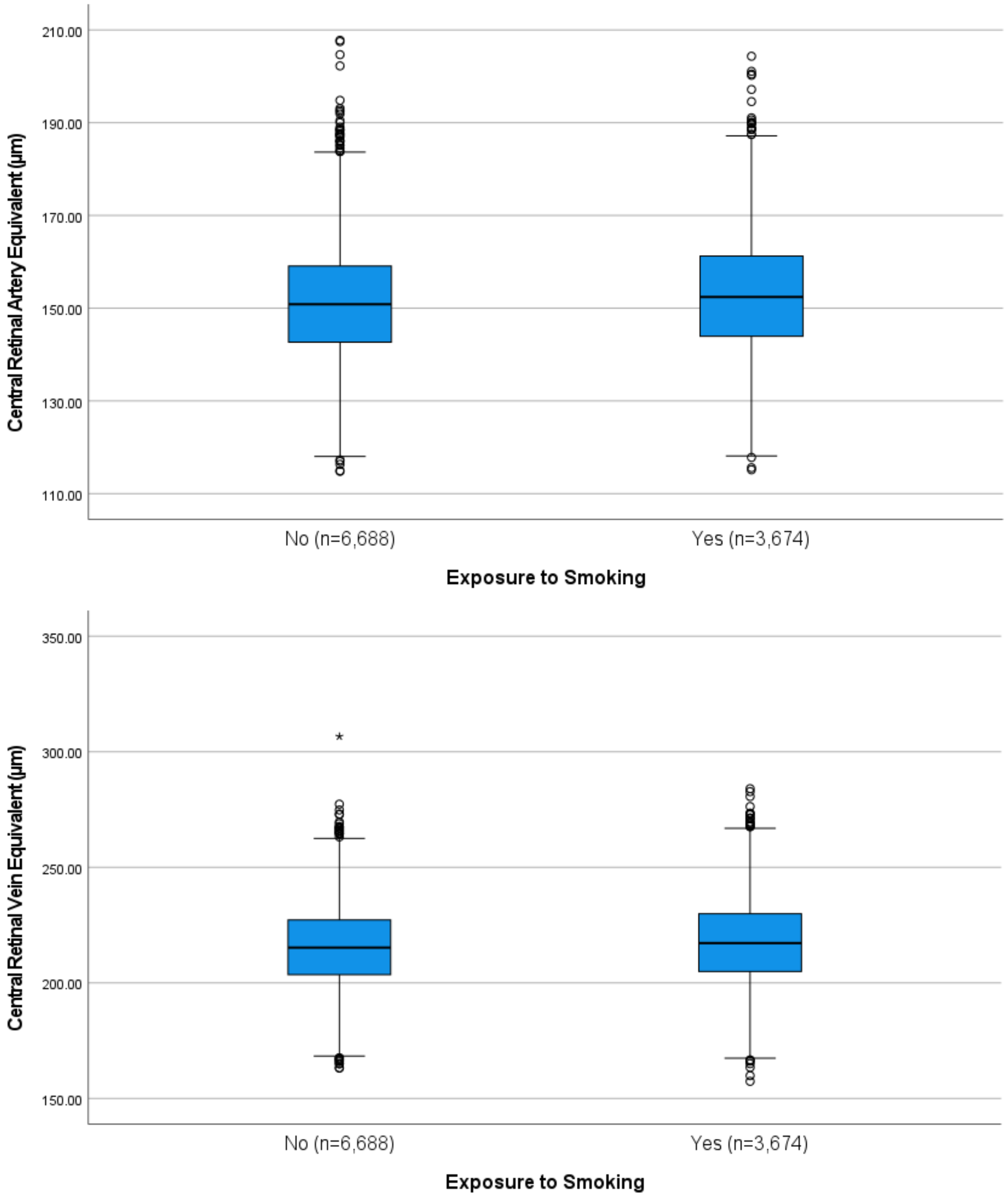

The horizontal line within the box represents the median value. The top end of the box represents the 75th quartile and the bottom of the box represents the 25th quartile. The whiskers end at the upper and lower adjacent values, the location of the furthest point that is within 1.5 IQRs of the first and third quartiles. Dots represent outside values.

Supplementary Figure 3. Subgroup Analysis Stratified by Systolic Blood Pressure (SBP), Sex, and Body Mass Index (BMI) for the Association between Exposure to Smoking and Retinal Vessel Calibers

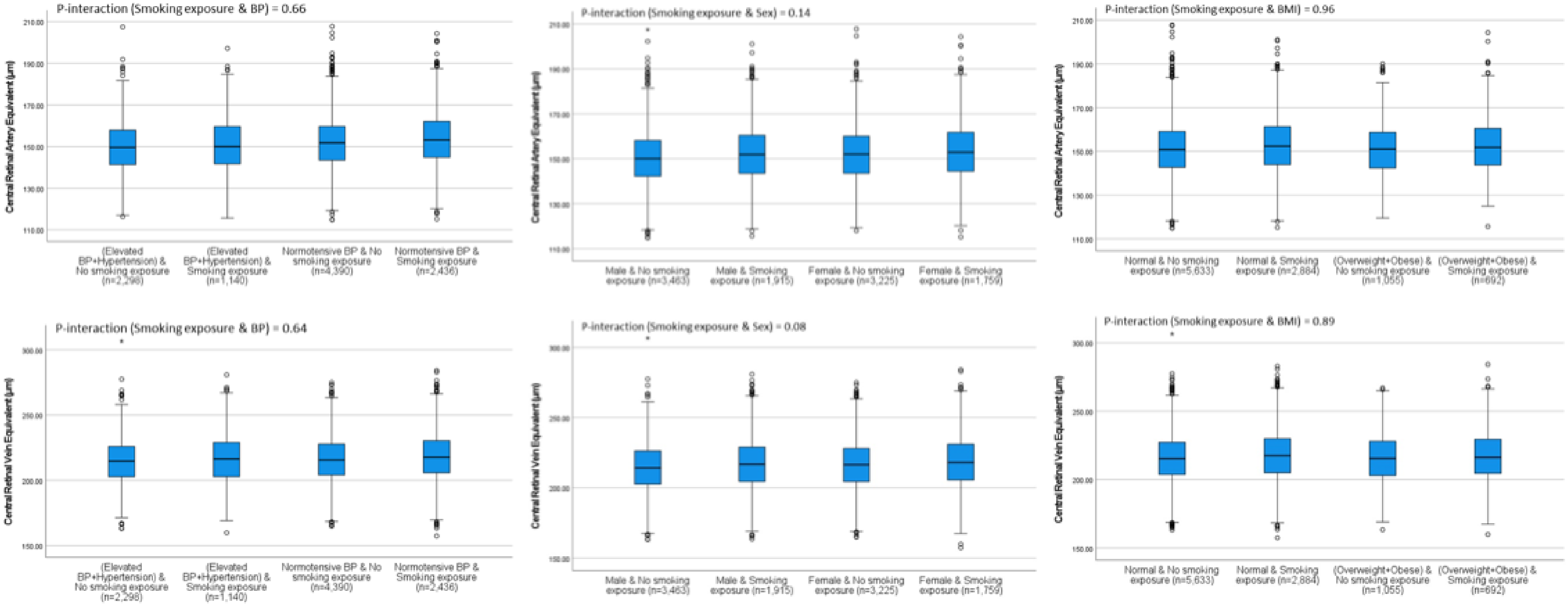

The horizontal line within the box represents the median value. The top end of the box represents the 75th quartile and the bottom of the box represents the 25th quartile. The whiskers end at the upper and lower adjacent values, the location of the furthest point that is within 1.5 IQRs of the first and third quartiles. Dots represent outside values.
